# The Effects of Padé Numerical Integration in Simulation of Conservative Chaotic Systems

**DOI:** 10.3390/e21040362

**Published:** 2019-04-03

**Authors:** Denis Butusov, Artur Karimov, Aleksandra Tutueva, Dmitry Kaplun, Erivelton G. Nepomuceno

**Affiliations:** 1 Youth Research Institute, Saint Petersburg Electrotechnical University “LETI”, Saint Petersburg 197376, Russia; 2 Department of Computer Aided Design, Saint Petersburg Electrotechnical University “LETI”, Saint Petersburg 197376, Russia; 3 Department of Automation and Control Processes, Saint Petersburg Electrotechnical University “LETI”, Saint Petersburg 197376, Russia; 4 Control and Modelling Group (GCOM), Department of Electrical Engineering, Federal University of São João del-Rei, São João del-Rei, MG 36307-352, Brazil

**Keywords:** Numerical integration, Padé approximation, nonlinear systems, dynamical chaos, computer simulation, ODE solvers

## Abstract

In this paper, we consider nonlinear integration techniques, based on direct Padé approximation of the differential equation solution, and their application to conservative chaotic initial value problems. The properties of discrete maps obtained by nonlinear integration are studied, including phase space volume dynamics, bifurcation diagrams, spectral entropy, and the Lyapunov spectrum. We also plot 2D dynamical maps to enlighten the features introduced by nonlinear integration techniques. The comparative study of classical integration methods and Padé approximation methods is given. It is shown that nonlinear integration techniques significantly change the behavior of discrete models of nonlinear systems, increasing the values of Lyapunov exponents and spectral entropy. This property reduces the applicability of numerical methods based on Padé approximation to the chaotic system simulation but it is still useful for construction of pseudo-random number generators that are resistive to chaos degradation or discrete maps with highly nonlinear properties.

## 1. Introduction

The influence of numerical methods on discrete models of chaotic systems is widely studied. While highly accurate numerical methods for chaotic problems integration have been recently developed [1,2], some studies reveal the negative aspects of popular discretization techniques [3,4] and discover the additional properties introduced by numerical errors [5]. Thus, when new class of integration methods appears, the collateral numerical effects are of certain interest.

A relationship between Padé approximation and numerical solution of an ordinary differential equation is well known [6,7]. From this point of view, various integration methods implement various types of approximation leading to different properties, such as the order of accuracy and A-stability [8]. Usually, the Padé approximant is contained in the numerical formula of the integration method indirectly. Meanwhile, direct expression of the increment function as a Padé approximant leads to a special class of nonlinear integration methods [9,10,11,12]. An analysis shows that these methods can demonstrate some new properties, inaccessible by linear one-step and multistep methods. The most promising application of these methods is the integration of problems with the singularity in the solution that can possess higher numerical efficiency in comparison to classical approaches [10,11,12]. Another attractive feature of these formulae is that they can achieve theoretical A-stability without implicit computations [13]. Although these methods deal well with singular nonlinear problems, no numerical evidence is found in the literature concerning their behavior in solving highly nonlinear ODEs, e.g., chaotic problems. When a continuous dynamical system is simulated using this technique, a presence of nonlinearity in the method itself can corrupt the original bifurcation pattern, phase space volume, and the other properties of the system. On the other hand, it may appear useful to avoid quasi-chaotic regimes, since some other nontrivial techniques are fruitful in dealing with this issue [14].

The aim of our study is to find out practical applicability of nonlinear integration technique based on direct Padé approximation to chaotic problems. The main motivation of the research is that the problem of finding a reliable numerical method for chaotic system simulation is still urgent in nonlinear science. Any type of discretization introduces undesirable nonlinear distortion in the differential equation solution [5]. To suppress the round-off and truncation errors, an extensive technique of extremely high order approximation and extended precision can be used [15], but long-term simulation by such methods can only be performed on supercomputers. If double precision is used and the truncation error is reduced by an appropriate method order selection below the numerical noise, the round-off error anyway influences the simulation results [16], so a reasonable tradeoff between round-off and truncation error may result in simulation time economy with minimal decrease in reliability. Thus, more suitable integration scheme can result in a notable increase in simulation performance. The conservative flows [17] are the most influenced by discretization among the chaotic systems due to their unique symmetrical and energy properties [18]. Therefore, we chose the conservative Nosé–Hoover system as a practical example for our study.

The last of the paper is organized as follows. In Section 2, integration methods, based on the direct Padé approximation of various orders, are introduced. In Section 3, bifurcation diagrams and Lyapunov spectrum are given for the Nosé–Hoover test chaotic system, and the results of long-term simulation analysis are described. We investigate the phase volume dynamics and calculate the spectral entropy including 2D dynamical maps for various discrete models of the test chaotic system. Finally, in Section 4 some conclusions are given.

## 2. Materials and Methods 

### 2.1. Integration Methods Based on Padé Approximants

For the initial value problem $\dot{y}=f\left(t,y\right)$ the numerical integration method is usually found in a form of a linear recurrence equation:

(1) $$y_{n+1}=y_{n}+h\Phi_{h}(t,y)$$

where Ф*_h_* denotes a so-called increment function. Digressing from this traditional approach, one can find a class of nonlinear methods based on a rational approximation of a form:

(2) $$y_{n+1}=y_{n}+\frac{h\Theta_{h}(t,y)}{\Phi_{-h}(t,y)}$$

where Θ*_h_* denotes a special numerator increment function providing a required order of accuracy. Up to date, several methods of a class (2) have been derived. The most popular one is a simple nonlinear method proposed by L. Wuytack, C. Brezinski and some other researchers [8,9]:

(3) $$y_{n+1}=y_{n}+\frac{hf^{2}(t_{n},y_{n})}{f(t_{n},y_{n})-\frac{h}{2}f^{\prime }(t_{n},y_{n})}$$

It is of order 2 and represents the simplest Padé approximation of the solution [9]. Here, $f^{\prime }$ stands for the derivative with respect to time, namely,

(4) $$f^{\prime }(t,y)=\frac{\partial f}{\partial y}(t,y)f(t,y)+\frac{\partial f}{\partial t}(t,y)$$

In many practical cases, Equation (4) can be solved analytically, while any approximation of order 1 will also yield the 2nd-order of accuracy to the resulting method. For the linear test problem, the method (3) is *A*-stable and explicit. For a vector problem, the division and power in (3) should be performed in an element-wise manner [12].

To derive some high-order methods of this type, several techniques have been developed recently [11,12,13]. Here we show one general approach providing a simple way to obtain the Padé integration method of arbitrary order. We consider only the approximations of a type [(*p*–1)/(*p*–1)], where *p* stands for the order of the resulting method.

Finding the derivatives of (2) and the derivatives of the corresponding Taylor series

$$x_{n+1}=x_{n}+h\Phi_{h}(t,x)$$

we require

$$x^{\left(k\right)}{}_{n+1}(0)=y^{\left(k\right)}{}_{n+1}(0)$$

for all natural *k* from 1 to the desired order *p*. The obtained formulas are summarized in Table 1. To construct the method of the required order, the numerator and denominator must contain a sum of the terms given in a table of all orders from 1 up to the required one. In this type of an approximant, *A*-stability of the resulting methods is provided for even orders. The *A*-stability for odd order approximants is missed as the power of *h* in the numerator is higher than in the denominator.

For example, the method of order 4 is given as follows

(5) $$y_{n+1}=y_{n}+\frac{hf_{n}^{2}-\frac{h^{3}}{12}(-4f_{n}^{\prime \prime }f_{n}+3{f_{n}^{\prime }}^{2})}{f_{n}-\frac{h}{2}f_{n}^{\prime }+\frac{h^{2}}{6}f_{n}^{\prime \prime }-\frac{h^{3}}{24}f_{n}^{\prime \prime \prime }}.$$

To construct methods of order higher than 6, one should notice in Table 1 the numerator coefficients are derivatives of (*y*^2^)^(*p*)^ by corresponding Taylor series multipliers, where terms containing *y_n_* are missed and odd derivatives of *y* are taken with a negative sign. The denominator represents the Taylor series for

$$(y(t_{n-1})-y_{n})/h$$

Since high order approximants contain high-order derivatives of the right-hand side function, they may be extremely complicated. Nevertheless, the success of Taylor series methods in some special problems motivates the further research of Padé methods.

### 2.2. Hypothesis

The hypothesis of this study is that Padé approximation methods can lead to finite-difference schemes with chaotic properties that are much different from schemes based on conventional methods. To explain the theory, we perform a brief sensitivity analysis here. We consider explicit second-order methods, namely Runge–Kutta 2 (explicit midpoint, EMP, or RK2) and Padé 2. The higher-order methods are omitted here, but the results for them are generally similar. Only autonomous IVPs $\dot{y}\left(t\right)=f\left(y\left(t\right)\right)$ are considered for simplicity, but the results persist for the time-dependent systems since only the sensitivity to phase variable perturbation is analyzed. The RK2 method is represented by the following equations.

(6) $$y_{n+\frac{1}{2}}=y_{n}+\frac{h}{2}f\left(y_{n}\right),$$

(7) $$y_{n+1}=y_{n}+hf\left(y_{n+\frac{1}{2}}\right).$$

Let the current point of the solution $y_{n}$ have a small perturbation ε, so that its actual value is ${\tilde{y}}_{n}=y_{n}+\varepsilon$. When the explicit Runge–Kutta 2 is applied, the following equations appear. From (6), we obtain

$${\tilde{y}}_{n+\frac{1}{2}}=y_{n}+\varepsilon +\frac{h}{2}f\left(y_{n}+\varepsilon \right)$$

which transforms after the Taylor expansion

$$f\left(y_{n}+\varepsilon \right)=f\left(y_{n}\right)+\varepsilon \;\frac{\partial f}{\partial y}\left(y_{n}\right)+O\left(\varepsilon^{2}\right)\approx \;f_{n}+\varepsilon \;f_{y}$$

to

(8) $${\tilde{y}}_{n+\frac{1}{2}}=y_{n}+\varepsilon +\frac{h}{2}\left(f_{n}+\varepsilon \;f_{y}\right)+O\left(\varepsilon^{2}\right)$$

In a similar manner, using (8), the equation (7) yields

$${\tilde{y}}_{n+1}=y_{n}+\varepsilon +hf_{n}+\varepsilon \;f_{y}+\frac{h^{2}}{2}f_{n}f_{y}+\frac{h^{2}f_{y}^{2}}{2}\varepsilon +O\left(\varepsilon^{2}\right)$$

So, the resulting perturbation after one integration step $\Delta y_{n+1}={\tilde{y}}_{n+1}-y_{n+1}$ is

(9) $$\Delta y_{n+1}=\;\varepsilon +\varepsilon \;f_{y}+\frac{h^{2}f_{y}^{2}}{2}\varepsilon +O\left(\varepsilon^{2}\right)$$

The same result is retained for arbitrary RK2 method. For the Padé 2 method the autonomous IVP, for which $f^{\prime }\left(y\right)=f_{y}f_{n}$, reads

(10) $$y_{n+1}=y_{n}+\frac{hf_{n}}{1-\frac{h}{2}f_{y}}$$

in case $f_{n}\neq 0$. Denote

(11) $$F\left(y_{n},h\right)=\frac{f_{n}}{1-\frac{h}{2}f_{y}}$$

Taylor expansion of (11) near zero gives

(12) $$F\left(y_{n},h\right)=F\left(y_{n},0\right)+h\frac{\partial F}{\partial h}\left(y_{n},0\right)+O\left(h^{2}\right)$$

Taking into account the relation $f_{y}(y_{n}+\varepsilon )=\;f_{y}+\varepsilon f_{yy}+O\left(\varepsilon^{2}\right)$ we obtain in a perturbed point $y_{n}+\varepsilon$ the following equation:

(13) $$\dot{F}\left(y_{n}+\varepsilon ,0\right)={{\left(\frac{f_{n}+\varepsilon f_{y}}{1-\frac{h}{2}\left(f_{y}+\varepsilon f_{yy}\right)}\right)}^{\prime }|}_{h=0}=\frac{\left(f_{n}+\varepsilon f_{y}\right)\left(f_{y}+\varepsilon f_{yy}\right)}{2}.$$

Substituting (13) into (12) and then (12) into (10), we eventually get

$${\tilde{y}}_{n+1}=y_{n}+\varepsilon +hf_{n}+\varepsilon \;f_{y}+\frac{h^{2}}{2}f_{n}f_{y}+\frac{h^{2}\left(f_{y}^{2}+f_{yy}f_{n}\right)}{2}\varepsilon +O\left(\varepsilon^{2}\right)$$

Therefore,

(14) $$\Delta y_{n+1}=\;\varepsilon +\varepsilon \;f_{y}+\frac{h^{2}\left(f_{y}^{2}+f_{yy}f_{n}\right)}{2}\varepsilon +O\left(\varepsilon^{2}\right)$$

The difference between (9) and (14) includes the term $h^{2}f_{yy}f_{n}\varepsilon /2$, which is absent in the Runge–Kutta 2 scheme. 

For any RK method in a point ${\tilde{y}}_{n+1}$

$${\tilde{y}}_{n+1}=\;y_{n}+\varepsilon +h\Phi \left(y_{n}+\varepsilon \right)$$

which, after Taylor expansion, produces

(15) $${\tilde{y}}_{n+1}=\;y_{n}+\varepsilon +h\left(\Phi \left(y_{n}\right)+\varepsilon \frac{\partial \Phi }{\partial y}\left(y_{n}\right)+\ldots \right)$$

On the other hand, any Padé method

$${\tilde{y}}_{n+1}=\;y_{n}+\varepsilon +\frac{h\Omega \left(y_{n}+\varepsilon \right)}{\Phi \left(y_{n}+\varepsilon \right)}$$

yields, considering the same Taylor expansion

(16) $${\tilde{y}}_{n+1}=\;y_{n}+\varepsilon +h\left(\frac{\Omega \left(y_{n}\right)}{\Phi \left(y_{n}\right)}+\varepsilon \frac{\frac{\partial \Omega }{\partial y}\left(y_{n}\right)\Phi \left(y_{n}\right)-\frac{\partial \Phi }{\partial y}\left(y_{n}\right)\Omega \left(y_{n}\right)}{\Phi^{2}\left(y_{n}\right)}+\ldots \right)$$

Comparing any methods of these types which possess equal accuracy order, one can see that (16) is more sensitive than (15) in case both partial derivatives in the numerator of (16) are nonzero.

## 3. Results

### 3.1. Bifurcation, Lyapunov Spectrum, and Spectral Entropy Analysis

Nosé [19] and Hoover [20] proposed the following conservative dynamical system [21].

(17) $$\begin{array}{l} \dot{x}=y;\\ \dot{y}=-x-ayz;\\ \dot{z}=b\left(y^{2}-1\right). \end{array}$$

**Figure 1:**
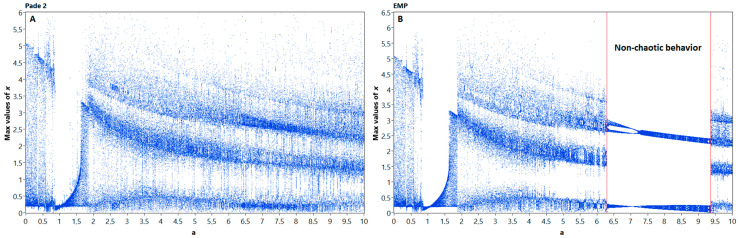
Bifurcation diagrams for Nosé–Hoover models, obtained by nonlinear Padé 2 method (**A**) and linear (**B**) EMP method. Note, that a nonchaotic area between *a* = 6.3 and *a* = 9.5 disappeared on the left image, which indicates the significant changes in system dynamics.

In model (17) we denote *a* and *b* as system parameters. We plotted bifurcation diagrams for two discrete models of Nosé–Hoover obtained by different discrete operators (see Figure 1). Among second-order integrators, we chose the explicit midpoint method and Padé 2 method (3). Fourth-order ODE solvers are represented by the explicit Runge–Kutta 4 (RK4) method [22] and Padé 4 method given by the formula (5). Simulation time was 500 s, with the transient time of 100 s, and integration step size was 0.01 sec. All the experiments were performed in NI LabVIEW 2018 64-bit simulation system on Core i5 processor with double floating point precision. Parameter *a* varied within the range *a* ∈ [0; 10] with 0.05 step. One can see that the model obtained by the Padé 2 method, demonstrates chaotic behavior for nearly all investigated values of *a*, and lacks the nonchaotic interval from *a* = 6 to *a* = 9.5, where all other methods exhibit regular oscillations.

**Figure 2:**
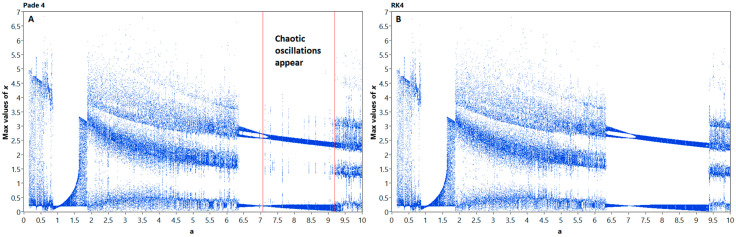
Bifurcation diagrams for Nosé–Hoover models, obtained by Padé 4 (**A**) and RK4 (**B**) methods, respectively. Note the artifacts appearing in Padé 4 diagram, which indicate the possible changes in system oscillations mode introduced by nonlinear integration technique.

The experimental results for fourth-order methods are shown in Figure 2. One can see that Padé 4 method introduces additional points that are observable on the bifurcation diagram.

**Figure 3:**
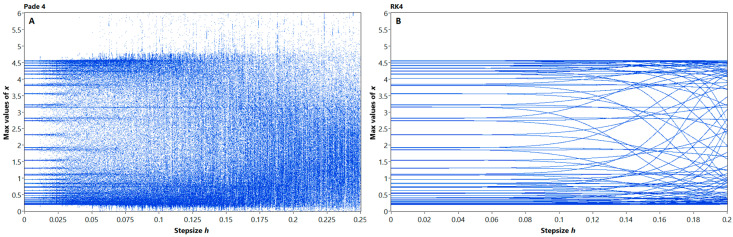
Step bifurcation diagrams for Nosé–Hoover models, obtained by Padé 4 (**A**) and RK4 (**B**) methods, respectively.

In computer simulations, the integration step size can also affect the behavior of the nonlinear models. To evaluate the impact of step size to the discrete models of system (17), we plotted step bifurcation diagrams, or *h*-diagrams (Figure 3) for Padé 4 and RK4 methods. Second-order methods provide stable solutions only for relatively small step sizes due to relative stiffness of the system, thus their *h*-diagrams are not representative. 

One can see from Figure 3 that the discrete models behave in a different manner. The model, obtained by RK4 method, only show phase shifts, while the oscillations mode remains nearly the same. The Padé 4 model is different: while the step size increases, the regime of oscillations unpredictably changes. This confirms the abovementioned increase in nonlinearity, which can be found in the bifurcation diagrams in Figure 1 and Figure 2. The depicted problem is complicated by the varying numerical stability of studied methods; therefore we chose the 0.01 s. and 0.005 s. fixed step sizes for experimental study, which guaranteed the stability of solutions for all investigated methods. 

**Figure 4:**
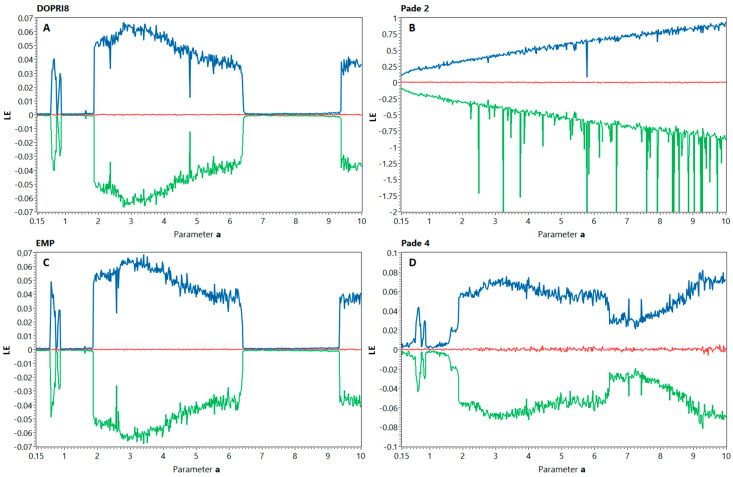
Lyapunov spectrum for discrete models of the Nosé–Hoover system obtained by the different numerical integration methods: DOPRI8 reference solution (**A**), Padé 2 method (**B)**, explicit midpoint method (**C**), and Padé 4 method (**D**).

However, the bifurcation analysis is only a qualitative tool, so we performed an additional quantitative study. One of the known ways to measure chaos in dynamical systems is the calculation of the Lyapunov spectrum [23]. The analysis of the spectrum reveals the symmetry of the system through the sum of all exponents, which is zero for conservative flows. Our hypothesis is that changes, introduced by nonlinear integration techniques into the Nosé–Hoover system, would appear in spectrum plot. As a reference solution, we chose the Lyapunov spectrum obtained from simulation by the Dormand–Prince method of order 8 (DOPRI8) with extended precision. Figure 4 represents the results for all investigated models: reference model by DOPRI8 and the studied models by Padé 2, Padé 4, and explicit midpoint method (EMP). Simulation time was set as 10^4^ s. with the transient time of 100 s, and integration step size was 0.005 s. Parameter *a* varied within the range *a* ∈ [0.1; 10] with a 0.05 step. One can see that reference spectrum clearly indicates the symmetry of the system and the sum of Lyapunov exponents is nearly zero. Some digital noise was introduced due to the finite precision. The spectrum of the model, obtained by Padé 2 method, shows the significant increase of nonlinearity as predicted in Section 2.2. The graph obviously lacks the nonchaotic “well” in the range *a* ∈ [6.25; 9.5], which can be observed in the reference method. Additional “splashes” indicate the moments of trajectory destruction and stability loss. The explicit midpoint method, also being a nonsymmetric integrator, does not show such behavior and we can only notice the disappearance of spikes in *a* = 4.85, which can be explained by round-off error. Another interesting phenomenon that remained undetected during the bifurcation analysis appears in Padé 4 model, which also tends to behave “more chaotic” than the reference model. Contrary to the reference method, the Padé 4 algorithm continuously increases the Lyapunov exponents, while *a* changes its value. Due to higher order of precision, we still can observe the “well” at *a* ∈ [6.25; 9.5] and relatively symmetric spectrum, but the sum of exponents is nonzero and second exponent is disturbed and obviously increases the value. The last is correct for Padé 2 method spectrum as well, but is not clearly visible due to the different scale.

Moreover, the behavior of discrete models obtained by linear methods seems to be less dependent on accuracy order contrary to the nonlinear techniques. 

To clarify the obtained results, we investigated the phase volume dynamics and spectral entropy of the discrete models over long simulation time.

### 3.2. Long-term Simulation and Phase Volume Dynamics

**Figure 5:**
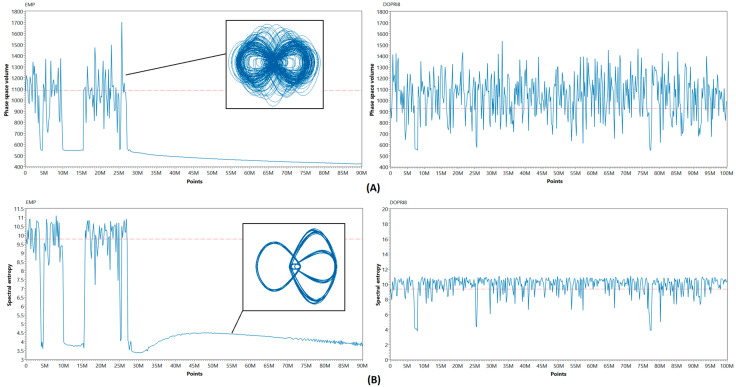
Phase space volume (**A**) and spectral entropy (**B**) dynamics for Nosé–Hoover models obtained by explicit Runge–Kutta methods. Note the chaos degradation in the explicit midpoint method (EMP) model. The Dormand–Prince 8 (DOPRI8) method is also prone to this defect, but manages to keep the solution chaotic over a relatively long simulation time due to high accuracy order.

In this section, we study the discretization effects appearing in computer models of chaotic systems during long-term simulations. It was previously shown [14] that every discrete system with finite data precision inevitably quits the chaotic regime of oscillations. We thus investigated the dynamics of the phase space volume for various discrete models of the Nosé–Hoover system. To obtain the phase volume plots, we calculated the changes in the volume of the system attractor with a 2000 s nonsliding window over long simulation time. We used the approximate algorithm and calculated the phase space volume as $V=\prod_{i=0}^{n}\left(\max\left(x_{i}\right)-\min\left(x_{i}\right)\right)$ with *n* = 3 for the Nosé–Hoover system. To prove the experimental results, we added the spectral entropy estimation, which corresponds with the existence of self-organizing or chaotic processes in the flow [24]. The simulation was performed with the integration step *h* = 0.01 s., initial conditions were [0.2; 0; 0.2], and nonlinearity parameters were chosen *a* = *b* = 0.7. The overall simulation time was 10^6^ s. Figure 5 represents the simulation results for models obtained by linear integration techniques, showing the difference between second-order EMP and eighth-order DOPRI8 method. One can see the good correspondence between phase space volume and spectral entropy plots.

The EMP method tends to shrink the phase space of the discrete model, driving the system into regular nonchaotic oscillations as the values of phase space volume and spectral entropy asymptotically decrease after 27M points. 

**Figure 6:**
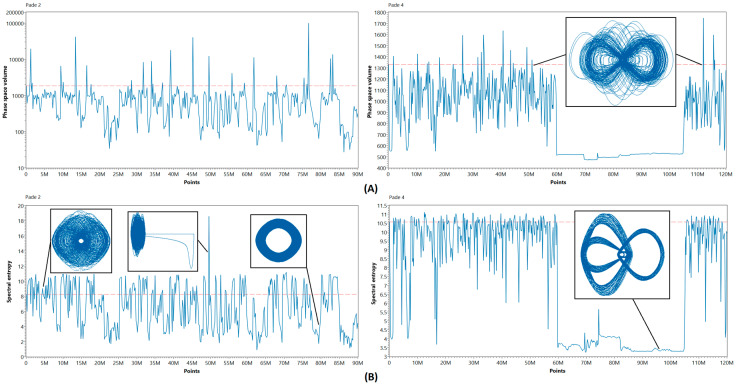
Phase space volume (**A**) and spectral entropy evolution (**B**), calculated for Nosé–Hoover system models obtained by Padé 2 and Padé 4 numerical methods.

Figure 6 illustrates the results for models obtained by nonlinear integration techniques. One can see that the solutions consequently pass through various chaotic regimes during the long-term simulation, exhibiting the phase transition behavior [25]. This variety of regimes may appear in DOPRI8 and EMP models only when values of nonlinearity parameters are changed. Thus, the nonlinear behavior of discrete models of Nosé–Hoover system is richer when they are obtained by numerical integration methods based on the direct Padé approximation. Another interesting observation one can see in a plot of the Padé 4 method. The chaos degradation occurs at 60M points, which resembles the behavior of EMP method. Nevertheless, the chaotic oscillations reappear at 105M, completely restoring the initial behavior of the system. Thus, we can conclude that the diversity of oscillation modes in the discrete models obtained by Padé approximation methods provides switching between chaotic and nonchaotic oscillations, major changes in the appearance of attractor (see Figure 6), and other nonlinear effects. The nature of these phenomena is obviously in the nonlinear properties of the discrete operators described in Section 2.2.

Finally, we need to answer one more question: how does the application of nonlinear integration techniques affect the parameters space of the system?

### 3.3. Dynamical maps

**Figure 7:**
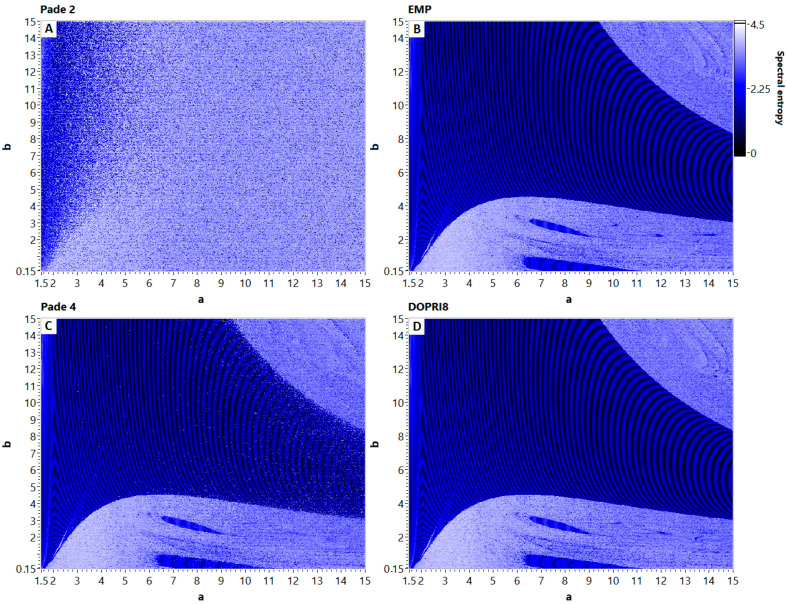
2D spectral entropy maps of discrete models of Nosé–Hoover system. Note the noise, appearing in Padé 4 map (**C**) and completely destructed map in Padé 2 case (**A**). The map, obtained for linear EMP method (**B**) is close to the reference DOPRI8 solution (**D**).

The 2D dynamical maps show how the behavior of the system depends on two nonlinearity parameters. Reconsidering all previously reported results, we plotted dynamical maps for four discrete models of the Nosé–Hoover chaotic system (Figure 7). Parameters *a* and *b* of the system (1) varied within the range [0.15; 15] in this experiment, and the spectral entropy value was plotted as a pixel color on the map. One can see that Padé methods change the regime of oscillations in the discrete Nosé–Hoover model for any selected values of nonlinearity parameters.

While the Padé 4 method exhibits only a slightly «noised» map, the application of Padé 2 method completely changes the properties of the resulting model, making the entire map covered by «chaotic sea». The maps obtained by linear integration techniques are independent from the method’s order of accuracy that confirms the results of one-dimensional bifurcation and long-term simulations analysis.

## 4. Conclusions & Discussion

In this paper, the nonlinear integration techniques based on a direct Padé approximation of the solution have been applied to the conservative nonlinear chaotic system. As a test problem, we chose the Nosé–Hoover system and performed a series of numerical experiments with the discrete models of the system. We have clearly shown that the nonlinearity inherent to Padé integration methods introduces valuable distortion in the properties of the chaotic system. To evaluate these distortions numerically, the Lyapunov spectrum, phase space volume, and spectral entropy dynamics during a long-term simulation were studied. Two discrete models obtained by conventional integration techniques were compared with models obtained by nonlinear techniques. We constructed bifurcation diagrams and 2D spectral entropy maps to investigate the possible variety of oscillation modes. Bifurcation diagrams show that chaotic behavior appears in models obtained by nonlinear integration techniques for those parameter values where chaos does not appear in conventional models. The estimation of the Lyapunov spectrum shows that the methods based on a direct Padé approximation tend to increase chaotic behavior, which expresses in larger values of Lyapunov exponents and their positive dynamics with the increase of nonlinearity parameters. The symmetry of the continuous prototype system is broken in discrete models obtained by nonlinear integration techniques. The long-term simulation shows sufficient changes of phase space volume and spectral entropy during simulation for all the investigated methods. Unlike the conventional methods, nonlinear integration methods did not show any tendency to the volume suppression or long-term chaos degradation. Instead, they show notable phase volume jittering and ability to recover from nonchaotic regimes after a sufficient time periods. The 2D spectral entropy maps discover the “blurring” effect of the Padé methods and the extension of chaotic areas comparing to reference model. The spectral entropy is sufficiently higher in discrete models obtained by nonlinear integration techniques that can be useful in cryptographic applications. 

To summarize, our experimental study revealed not only the limitations of numerical methods based on a direct Padé approximation when simulating chaotic systems, but also opened the possibilities to construct highly nonlinear and less predictable discrete chaotic maps. Obtained models can be used as testbench systems in various statistical studies [25] or for simulation of nonstationary processes with multifractal properties. Though an accurate simulation with nonlinear integration techniques meets significant difficulties, these methods can improve the algorithms of pseudo-random number generators [26,27], making them able to avoid quasi-chaotic regimes during long-term runs. They also can provide improved topological mixing and diffusion properties required in chaos-based cryptosystems [28].

## Figures and Tables

**Table 1:** Terms of Padé methods of different accuracy order.

Accuracy Order	Numerator	Denominator
1	$-hf_{n}^{2}$	$-f_{n}$
2		$\frac{h}{2}f_{n}^{\prime }$
3	$\frac{h^{3}}{12}(-4f_{n}^{\prime \prime }f_{n}+3{f_{n}^{\prime }}^{2})$	$-\frac{h^{2}}{6}f_{n}^{\prime \prime }$
4		$\frac{h^{3}}{24}f_{n}^{\prime \prime \prime }$
5	$\frac{h^{5}}{360}(-6f_{n}^{\left(4\right)}f_{n}-10{f_{n}^{\prime \prime }}^{2}+15f_{n}^{\prime \prime \prime }f_{n}^{\prime })$	$-\frac{h^{4}}{120}f_{n}^{\left(4\right)}$
6		$\frac{h^{5}}{720}f_{n}^{\left(5\right)}$

## References

[1] Lozi R., Pchelintsev A.N. (2015). A new reliable numerical method for computing chaotic solutions of dynamical systems: The Chen attractor case. Int. J. Bifurc. Chaos.

[2] Lozi R., Pogonin V.A., Pchelintsev A.N. (2016). A new accurate numerical method of approximation of chaotic solutions of dynamical model equations with quadratic nonlinearities. Chaos Solitons Fract..

[3] Corless R.M., Essex C., Nerenberg M.A.H. (1991). Numerical methods can suppress chaos. Phys. Lett. A.

[4] Lozi R., Letellier C., Gilmore R. (2013). Can we trust in numerical computations of chaotic solutions of dynamical systems?. Topology and Dynamics of Chaos.

[5] Nepomuceno E.G., Mendes E.M. (2017). On the analysis of pseudo-orbits of continuous chaotic nonlinear systems simulated using discretization schemes in a digital computer. Chaos Solitons Fract..

[6] Wanner G., Hairer E., Nørsett S.P. (1978). Order stars and stability theorems. BIT Numer. Math..

[7] Alaybeyi M. (1994). On the Relationship between Integration and Padé Approximation. Ph.D. Thesis.

[8] Brezinski C., Van Iseghem J. (1994). Padé approximations. Handbook of Numerical Analysis.

[9] Wuytack L. (1975). Numerical integration by using nonlinear techniques. J. Comput. Appl. Math..

[10] Werner H., Wuytack L. (1978). Nonlinear quadrature rules in the presence of a singularity. Comput. Math. Appl..

[11] Ramos H. (2007). A non-standard explicit integration scheme for initial-value problems. Appl. Math. Comput..

[12] Gadella M., Lara L.P. (2013). A Numerical method for solving ODE by rational approximation. Appl. Math. Sci..

[13] Ramos H., Singh G., Kanwar V., Bhatia S. (2017). An embedded 3 (2) pair of nonlinear methods for solving first order initial-value ordinary differential systems. Numer. Algorithms.

[14] Karimov T.I., Butusov D.N., Pesterev D.O., Predtechenskii D.V., Tedoradze R.S. Quasi-chaotic mode detection and prevention in digital chaos generators. Proceedings of the IEEE Conference of Russian Young Researchers in Electrical and Electronic Engineering (ElConRus).

[15] Liao S.J., Wang P.F. (2014). On the mathematically reliable long-term simulation of chaotic solutions of Lorenz equation in the interval [0,10000]. Sci. China Phys. Mech..

[16] Grazier K.R., Newman W.I., Hyman J.M., Sharp P.W., Goldstein D.J. (2005). Achieving Brouwer’s law with high-order Stormer multistep methods. ANZIAM J..

[17] Jafari S., Sprott J.C., Dehghan S. (2019). Categories of conservative flows. Int. J. Bifurc. Chaos.

[18] Huynh V.V., Ouannas A., Wang X., Pham V.-T., Nguyen X.Q., Alsaadi F.E. (2019). Chaotic map with no fixed points: entropy, implementation and control. Entropy.

[19] Nosé S. (1984). A molecular dynamics method for simulations in the canonical ensemble. Mol. Phys..

[20] Hoover W.G. (1985). Canonical dynamics: equilibrium phase-space distributions. Phys. Rev. A.

[21] Pham VT., Volos C., Kapitaniak T. (2017). Systems without equilibrium. Systems with Hidden Attractors.

[22] Hairer E., Norsett S.P., Wanner G. (1993). Solving Ordinary Differential Equations I: Nonstiff Problems.

[23] Wolf A., Swift J.B., Swinney H.L., Vastano J.A. (1985). Determining Lyapunov exponents from a time series. Physica D.

[24] Crepeau J., Lavar K.I. (1990). On the spectral entropy behavior of self-organizing processes. J. Non-Equilib. Thermodyn..

[25] Pyko N.S., Pyko S.A., Markelov O.A., Karimov A.I., Butusov D.N., Zolotukhin Y.V., Uljanitski Y.D., Bogachev M.I. (2018). Assessment of cooperativity in complex systems with non-periodical dynamics: Comparison of five mutual information metrics. Physica A.

[26] Tsai C., Wang H., Wu J. (2019). Three techniques for enhancing chaos-based joint compression and encryption schemes. Entropy.

[27] Kaya D., Ergun S. (2018). An analysis of deterministic chaos as an entropy source for random number generators. Entropy.

[28] Alvarez G., LI S. (2006). Some basic cryptographic requirements for chaos-based cryptosystems. Int. J. Bifurc. Chaos.

